# Characterization of a Nattokinase from the Newly Isolated Bile Salt-Resistant *Bacillus mojavensis* LY-06

**DOI:** 10.3390/foods11162403

**Published:** 2022-08-10

**Authors:** Yuan Li, Xiyu Tang, Liangqi Chen, Xinran Xu, Jinyao Li

**Affiliations:** 1 Institute of Materia Medica, Xinjiang University, Urumqi 830017, China; 2 Xinjiang Key Laboratory of Biological Resources and Genetic Engineering, College of Life Science and Technology, Xinjiang University, Urumqi 830017, China

**Keywords:** *Bacillus* nattokinase, fermentation process, heterologous expression, enzymatic properties, bioinformatics analysis, productivity analysis

## Abstract

Nattokinase is a potential new thrombolytic drug because of its strong thrombolytic effect, high safety, and low cost. However, there is no research reporting on bile salt-tolerant nattokinase-producing probiotics. In this study, the bile salt-tolerant nattokinase-producing strain *Bacillus mojavensis* LY-06 was isolated from local Xinjiang douchi, and the fermentation yield of nattokinase of 1434.64 U/mL was obtained by both a single factor experiment and an orthogonal experiment. A gene responsible for fibrinolysis (*aprY*) was cloned from the genome of strain *Bacillus mojavensis* LY-06, and the soluble expression of this gene in *Escherichia coli* (rAprY, fused with His-tag at C-terminus) was achieved; molecular docking elucidates the cause of insoluble expression of rAprY. The optimal pH and temperature for the fibrinolysis activity of nattokinase AprY fermented by *Bacillus mojavensis* LY-06 were determined to be pH 6.0 and 50 °C, respectively. However, the optimal pH of rAprY expressed in *Escherichia coli* was 8, and its acid stability, thermal stability, and fibrinolytic activity were lower than those of AprY. Bioinformatics analysis found that the His-tag carried at the C-terminus of rAprY could affect its acidic stability by changing the isoelectric point and surface charge of the enzyme; in contrast to AprY, changes in the number of internal hydrogen bonds and the flexibility of the loop region in the structure of rAprY resulted in lower fibrinolytic activity and poorer thermal stability.

## 1. Introduction

Cardiovascular disease is a serious threat to human health [1]. In cardiovascular disease, cerebral infarction, ischemic stroke, and myocardial infarction are all related to thrombus formed by fibrin and platelet coagulation, while thrombolytic drugs represented by urokinase, tissue plasminogen activator (t-PA), and streptokinase all have side effects such as severe bleeding or gastric ulcer [2,3]. Nattokinase is an alkaline serine protease with strong fibrinolytic and thrombolytic activity secreted by *Bacillus* natto, discovered in natto, the traditional Japanese fermented food, by Sumi et al. [4]. Clinical trials show that oral administration of nattokinase has the ability to reduce carotid plaque area and common carotid artery media thickness in patients with hyperlipidemia [5]. Nattokinase is considered to play a thrombolytic role through blood absorption after oral administration, but due to the large molecular weight of nattokinase, some studies also believe that nattokinase cannot be absorbed through the gastrointestinal tract [6]. The acid stability of nattokinase is poor, and the encapsulation of nattokinase with chitosan/casein-based microparticles is considered to be an effective method to overcome the weakness of nattokinase [7]. Nattokinase can not only directly hydrolyze thrombogenic amino acids and small peptides, but also participate in thrombolysis through a variety of indirect mechanisms, including the activation of prourokinase and tissue plasminogen activator (t-PA) to activate plasminogen [8,9,10], the reduction of plasma coagulation factor VII and coagulation factor VIII concentrations [11], the prevention of thromboxane formation [12], the downregulation of reactive oxygen species (ROS) production, and the activation of nuclear factor-κB (NF-κB), etc. [13]. Compared with traditional thrombolytic drugs, nattokinase has a relatively low administration risk, a large tolerable dose, and no side effects, such as gene mutation and chromosomal aberration, making it a new thrombolytic drug with great application potential [14,15].

Except for a few reports confirming that some marine *Bacillus subtilis* [16] and *Pseudomonas* sp. [17] can synthesize nattokinase, *Bacillus* sp. is the main strain for synthesizing nattokinase. At present, *Bacillus* synthesizing nattokinase has been successfully isolated from various fermented foods, including natto, tempeh, Chinese soybean paste, and chungkookjang [18,19,20,21]. At present, there is a lack of systematic research on the tolerance of nattokinase to the digestive system, and the development of probiotic products that synthesize nattokinase is an effective means to solve such digestive problems. However, there is still no report on *Bacillus* with high nattokinase production as an oral probiotic. Determining whether *Bacillus* can synthesize highly active nattokinase in vivo is the key to solving the problems resulting from a variety of unfavorable factors in the digestive tract environment (gastric acid, bile salts, protease, etc.) which have negative effects on the reproduction of *Bacillus* and their production of nattokinase [22]. Therefore, the development of nattokinase synthetic probiotics and their expressed nattokinase, with resistance to digestive tract system, has practical significance for the development of oral nattokinase.

Optimizing the fermentation process of *Bacillus* to synthesize nattokinase and realizing the high expression of nattokinase in engineering bacteria are both important ways to improve the yield and activity of nattokinase. Soybean, chickpea, and wheat bran are generally used as raw materials for the production of nattokinase by solid-state fermentation of *Bacillus* [20]. The by-product poly-L-glutamic acid produced in solid-state fermentation has been shown to increase the fermentation yield of nattokinase, but it also increases the risk of allergy [23,24]. The nattokinase liquid fermentation method overcomes the shortcomings of the solid-state fermentation method, such as low water content, poor fluidity, and difficulty in process monitoring, and is widely used in the industrial production of nattokinase [25,26]. At present, nattokinase has been expressed in *Escherichia coli* [27], *Lactobacillus* [28], *Bacillus* [29], and *Pichia pastoris* [30]. *Escherichia coli* is the most widely studied host for nattokinase expression. However, the recombinant nattokinase expressed in *Escherichia coli* exhibits problems such as low enzymatic activity and less soluble expression, and the comparative study of enzymatic properties compared with the natural nattokinase synthesized by *Bacillus* is lacking [31,32,33].

In this study, a bile salt-tolerant and nattokinase-producing *Bacillus mojavensis* LY-06 was screened from the fermented food douchi, and the fermentation process of nattokinase from *Bacillus mojavensis* LY-06 (AprY) was optimized by single factor and orthogonal experiments. According to the homology of the nattokinase structural gene, the nattokinase encoding gene of *Bacillus mojavensis* LY-06 (*aprY*) was amplified, and the active expression of recombinant AprY (rAprY, fused with His-tag at C-terminus) in *Escherichia coli* was realized. The enzymatic properties and productivity of AprY and rAprY were analyzed, respectively.

## 2. Materials and Methods

### 2.1. Samples, Plasmids, and Chemicals

Douchi samples were obtained from farmers’ markets in Xinjiang. *Escherichia coli* strains DH5α and BL21 (DE3) were used for the plasmid amplification and protein expression, respectively. The plasmid peT28a(+) was used for the induced expression. The DNA polymerase for PCR, T4 DNA ligase, and restriction enzymes were purchased from TaKaRa Co., Ltd. (Tokyo, Japan). Bovine bile salts, fibrinogen, and thrombin were purchased from Shanghai yuanye Bio-Technology Co., Ltd. (Shanghai, China). 

### 2.2. Assay of Fibrinolytic Activity

The fibrinolytic activity determination related to strain screening and fermentation process optimization were accomplished by using the fibrin plate method [34]. A total of 4 mg/mL fibrinogen (in 37 °C water bath), 50 U/mL thrombin, and 1% agarose (in a 60 °C water bath) were prepared using sodium barbital buffer (10.1 g/L barbital sodium, 7.4 g/L NaCl, 1 g/L gelatin, pH 7.8). A total of 7.5 mL of 1% agarose solution was mixed with 7.5 mL of 4 mg/mL fibrinogen solution and 0.4 mL of 50 U/mL thrombin solution, and the mixture was poured into the plate and left at room temperature for 30 min. A total of 1 μL of the nattokinase fermentation broth supernatant was added to the above plate, and the diameter of the transparent circle (mm^2^) was calculated after culturing at 37 °C for 18 h; the fibrinolytic activity was measured using a urokinase standard as a control.

The UV spectrophotometer method was used to characterize the enzymatic properties of AprY and rAprY (http://j-nattokinase.org/jnka_nk_english.html, accessed on 6 July 2022). A total of 0.4 mL of 0.72% (*w*/*v*) fibrinogen solution was mixed with 1.4 mL of 50 mM Tris-HCl (pH 8.0) buffer, and was incubated at 37 °C for 5 min. Then, 0.1 mL of thrombin solution (20 U/mL) was added to the above reaction system and was incubated at 37 °C for 10 min, then 0.1 mL of diluted enzyme solution was added to fully homogenize the mixture, and it was incubated in a 37 °C water bath for 60 min. After the reaction was stopped by adding 2 mL of 0.2 mM trichloroacetic acid and centrifuged at low speed, the absorbance at 275 nm of the supernatant was measured. One unit of enzyme activity (FU) is defined as the amount of enzyme required to change the absorbance at 275 nm by 0.01 per minute at 37 °C, pH 8.0.

### 2.3. Isolation of Strains Producing Nattokinase

Various douchi samples were purchased from farmers’ markets (Urumqi, China). Two grams of douchi were mixed with 5 mL sterilized normal saline and heated to 90 °C for 15 min to remove non-spore bacteria [35]. The supernatant was then collected and diluted in 0.9% sterile normal saline, plated onto the LB medium with 0.5% bile salts and 1.8% agar, and incubated at 37 °C for 24 h. Strains were separated and purified, then cultivated in 25 mL Erlenmeyer flasks with 5 mL of LB medium at 37 °C for 12 h in an orbital incubator at 220 rpm (OD_600_ = 1.5–1.6). We added 2% (*v*/*v*) inoculum culture (OD_600_ = 1.5–1.6) to 250 mL Erlenmeyer flasks containing 50 mL of LB medium and incubated these at 37 °C for 48 h in an orbital incubator at 220 rpm. Cultures were then centrifuged at 5000× *g* for 10 min, and the fibrin plate method was used to determine the thrombolytic activity of nattokinase in the fermentation supernatant. 

### 2.4. Identification of Strains Producing Nattokinase

The total genomic DNA of the bile salt-tolerant nattokinase-producing strain was extracted with a TIANamp Bacteria DNA Kit (Tiangen, Beijing, China). The bile salt-tolerant nattokinase-producing strain (*Bacillus mojavensis* LY-06) was further identified by gram staining and 16S rDNA PCR method using primer 27F (5′-AGAGTTTGATCCTGGCTCAG-3′) and 1492R (5′-TACGACTTAACCCCAATCGC-3′). PCR program was set as follows: 94 °C for 5 min; 94 °C for 30 s, 55 °C for 30 s, and 72 °C for 1 min, 30 cycles; 72 °C for 10 min and 4 °C preservation. PCR products were analyzed with agarose gel electrophoresis at 80 V and sequenced. Sequence homology was evaluated with nucleotide BLAST (BLASTN, NCBI), and a phylogenetic tree was constructed using MEGA X software. The screened colony was named *Bacillus mojavensis* LY-06.

### 2.5. Stress Tolerance Detection of Isolated Bacillus mojavensis LY-06 Strain

The *Bacillus mojavensis* LY-06 strain, which was screened in this study with bile salt tolerance and high yield of nattokinase, was inoculated into LB liquid medium at an inoculum of 2% (*v*/*v*), treated with 60, 70, and 80 °C water baths for 10 min, and cultured at 37 °C for 14 h. The growth of the strain was determined by detecting the OD_600_ of the bacterial medium. 

Test of acid tolerance: The strains were inoculated into LB liquid medium with different pH (5–10) according to the inoculum amount of 2% (*v*/*v*), and the OD_600_ of the bacterial liquid was recorded at corresponding time intervals.

Test of bile salt tolerance: The strains were inoculated into LB liquid medium with different concentrations of bovine bile salt (0.1–1.5%) according to the inoculation amount of 2% (*v*/*v*), and the OD_600_ of the bacterial liquid was recorded at corresponding time intervals.

### 2.6. Growth Curve Determination of Bacillus mojavensis LY-06 Strain

The *Bacillus mojavensis* LY-06 strain culture was inoculated into 20 mL of LB medium with 1% (*v*/*v*) inoculum, and cultivated at 37 °C for 14 h. The LB liquid medium without the test bacterial solution was used as a blank control, and its OD_600_ was measured at corresponding time intervals. 

### 2.7. Optimization of the Fermentation Process of AprY Production

The nattokinase fermentation broth (tryptone 12 g/L, glycerol 0.2%, K_2_HPO_4_ 2.3 g/L, KH_2_PO_4_ 12.5 g/L, CaCl_2_ 0.2 g/L, MgCl_2_ 0.69 g/L, sucrose 24 g/L, pH 7.2) was used as the original medium for nattokinase production. Different types of medium carbon sources (24 g/L of lactose, sucrose, glucose, and yeast extract), nitrogen sources (12 g/L of soybean flour, tryptone, soybean meal, and peptone), different concentrations of glycerol (2 g/L, 4 g/L, 6 g/L, 8 g/L, 10 g/L), CaCl_2_ (0.1 g/L, 0.2 g/L, 0.3 g/L, 0.4 g/L, 0.5 g/L), MgCl_2_ (0.1 g/L, 0.2 g/L, 0.3 g/L, 0.4 g/L, 0.5 g/L), MnSO_4_ (10^−3^ mol/L, 10^−4^ mol/L, 10^−5^ mol/L), and K_2_HPO_4_:KH_2_PO_4_ (0.1 g/L, 0.2 g/L, 0.3 g/L, 0.4 g/L, 0.5 g/L) were tested to determine the impact of AprY fermentation. The optimal fermentation broth conditions for the production of nattokinase by *Bacillus mojavensis* LY-06 strain were determined by orthogonal experiments on the factors that had a greater influence on AprY (Table 1).

### 2.8. Construction of the Expression Plasmids Encoding aprY in Escherichia coli

The coding sequence of AprY (*apr*Y) was cloned into the peT28a(+) vector for expression in *Escherichia coli* BL21 (DE3). The open reading frame of AprY was amplified from the total genomic DNA of strain *Bacillus mojavensis* LY-06 by PCR, using forward (5′-TGTGGATCCgtgagaagcaaaaaattgtggatca-3′) primers and reverse primers (5′-ccgCTCGAGttgtgcagctgcttgtacgt-3′) with BamHI and XhoI sites. The recombinant strain was selected by resistance to kanamycin.

### 2.9. Bacterial Expression and Purification of rAprY

The expression and purification method of rAprY refers to the previous research of Weng et al., with a little modification [36]. *Escherichia coli* BL21 (DE3) cells that carried the pET-28a-aprY expression vector were grown in LB medium that contained 50 mg/mL kanamycin at 37 °C, and rAprY overexpression was induced by adding IPTG to a final concentration of 0.1 mM when OD_600_ reached 0.7–0.8. The cells were further grown for 20 h at 18 °C and harvested by centrifugation. The bacterial pellet was re-suspended by adding PBS buffer (50 mM NaH_2_PO_4_, 300 mM NaCl, 10 mM imidazole, 5% glycerol, pH 7.4). The harvested cells were lysed by sonication and centrifuged to remove the cell debris. The suspension was centrifuged at 20,000× *g* for 30 min at 4 °C, and the supernatant was applied for subsequent purification carried out at 0–4 °C. The rAprY variants were purified by techniques of column chromatography using Ni-NTA column (Invitrogene, Carlsbad, CA, USA) and DEAE Sepharose Fast Flow column (Amersham Biosciences, Piscataway, NJ, USA). After the purification to homogeneity, the protein concentration was determined by the BCA protein assay reagent kit (Pierce).

### 2.10. Biochemical Characterization of AprY and rAprY

Purified AprY was obtained by ammonium sulfate precipitation and dialysis. The eluted fractions were assayed for enzyme activity, and the fractions were combined and concentrated. The fibrinolytic activity of purified nattokinase was tested using the UV spectrophotometer method [37]. The optimum pH of AprY and rAprY activity was determined using fibrin as a substrate in 100 mM sodium acetate buffer (pH 4.0–6.0), 100 mM phosphate buffered saline buffer (PBS buffer, pH 7.0–8.0), and 100 mM glycine NaOH buffer (pH 9.0–11.0). The effect of pH on AprY and rAprY stability was measured by placing the enzyme in buffers of different pH on ice for 30 min. The optimal temperature was detected by performing the standard assay at temperatures that ranged from 0 to 80 °C in PBS buffer (pH 8.0). The effect of temperature on AprY and rAprY stability was measured by placing the enzyme in a water bath at different temperatures for 30 min. 

### 2.11. Bioinformatics Analysis

The protein sequences of rAprY were submitted to the I-Tasser workplace, which built relatively accurate structural models (https://zhanggroup.org/I-TASSER/, accessed on 7 July 2022) [38]. The visualization of enzyme structures was conducted using PymoL software. Salt bridges and hydrogen bonds were calculated by ESBRI [39] and DSSP [40], respectively. Surface protein hydrophobicity was calculated using the ProtScale tool of Expasy (https://web.expasy.org/protscale/, accessed on 7 July 2022). The pKa value of residuals was calculated by H++ (http://newbiophysics.cs.vt.edu/H++/uploadpdb.php, accessed on 7 July 2022). The Perdy Flexy server (https://www.dsimb.inserm.fr/dsimb_tools/predyflexy/index.html, accessed on 7 July 2022) was used for calculating B-factor and RMSF of protein amino acid residues.

### 2.12. Productivity Curves of AprY and rAprY

To obtain the productivity curves at optimum pH and optimum temperature in the absence and presence of bile salts for AprY and rAprY: 0.3% bile salt was added to the reaction system containing enzyme and fibrin, and incubated for 300 min under optimal conditions of each enzyme (For AprY, 50 °C, pH 6; for rAprY, 50 °C, pH 8), and the free amino acids produced by the hydrolysis of fibrin was measured by the UV spectrophotometer method every 30 min. The enzyme reaction system without bile salt was used as the blank control group.

To obtain the productivity curves at high temperature in the presence of bile salts for AprY and rAprY: 0.3% bile salt was added to the reaction system containing enzyme and fibrin, and incubated for 300 min under 50 °C and 75 °C, respectively, and the free amino acids produced by the hydrolysis of fibrin was measured by the UV spectrophotometer method every 30 min. 

### 2.13. Statistical Analyses 

All samples were analyzed in triplicate, and the data were presented as the mean ± the standard deviation for each sample point. All data were collected to analyze the variance at *p* < 0.05, and Duncan’s multiple range test was applied to compare the mean values.

## 3. Results 

### 3.1. Screening and Identification of Bile Salt-Tolerant and High-Yielding Nattokinase Strains

**Figure 1:**
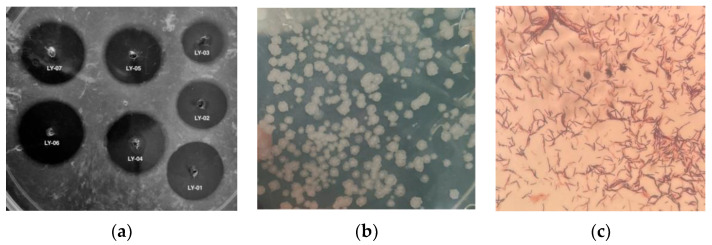
Screening of LY-06 strain using the fibrin plate method (**a**), morphological observation (**b**), and Gram staining (**c**).

Traditional antithrombotic drugs have shortcomings such as poor thrombolytic effects, bleeding tendency, and a single mode of administration. Nattokinase, which has high thrombolytic activity, few side effects, and can be administered orally, has become a potential new thrombolytic drug [35]. Nattokinase is mainly produced by the fermentation of *Bacillus*; some marine *Bacillus subtilis* [16] and *Pseudomonas* [17] have also been found to produce nattokinase. Although nattokinase has been isolated from a variety of fermented foods, there is no report on the growth of oral nattokinase probiotics in a bile salt environment until now, which limits the market application of nattokinase. In this study, douchi samples were subjected to a high temperature water bath and streaked on LB plates, and a total of 32 candidate *Bacillus* strains were isolated and purified. Through bile salt plate screening of these 32 strains, only 7 strains were found to grow normally, namely LY-01 to LY-07, respectively. Among these, only the LY-06 strain produced colonies after 24 h of culture, and the other 6 strains produced colonies after 48 h. Using the fibrin plate method, it was determined that the LY-06 strain had the greatest fibrinolytic activity (the area of the fibrinolytic transparent circle was 423 mm^2^) (Figure 1a). Therefore, we selected the LY-06 strain for follow-up experiments.

**Figure 2:**
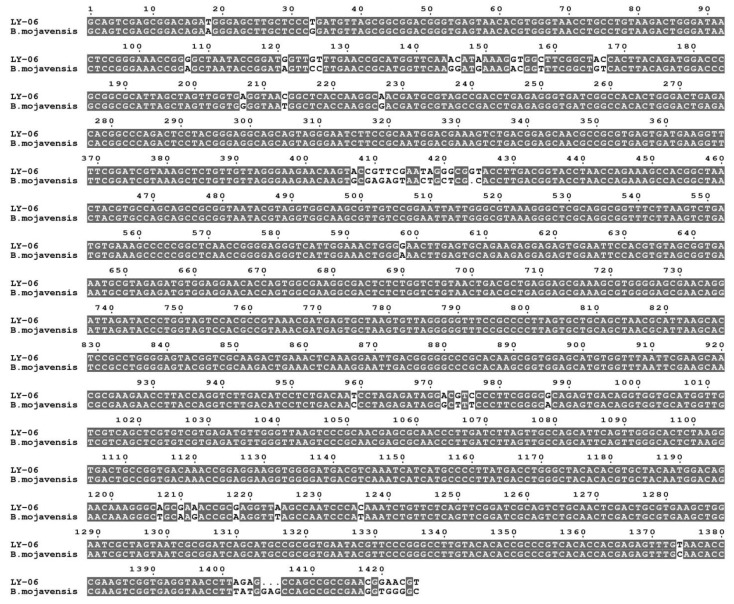
Multiple sequence alignment of LY-06 16S rDNA compared with *Bacillus mojavensis* ifo 15718 16S rDNA. LY-06: LY-06 16S rDNA; *Bacillus mojavensis*: *Bacillus mojavensis* ifo 15718 16S rDNA.

**Figure 3:**
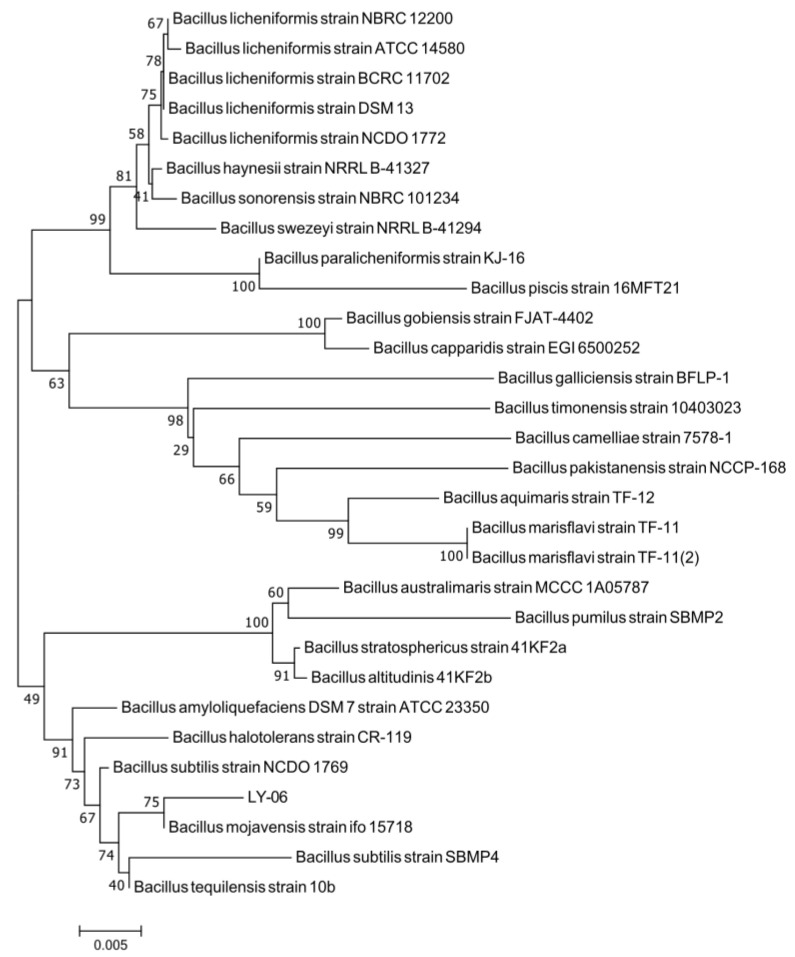
16S rDNA phylogenetic trees indicating the relations of strain LY-06 with the related organisms. The scale bar represents 0.005 nucleotide substitution per position.

We carried out morphological observation, Gram staining, and molecular biological identification of the LY-06 strain to determine the species of this strain. The colonies of LY-06 were flat and rough, with irregular edges, and could be stained purple-red with Gram dyes (Figure 1b,c). The 16S rDNA sequence of the total DNA of strain LY-06 was amplified using the 27F/1492R universal primers. The amplified fragments of about 1500 bp were sequenced, and the sequencing results were subjected to NCBI sequence alignment and phylogenetic tree construction. It was found that LY-06 has high homology with multiple *Bacillus* 16S rDNA fragments, and it is clustered with *Bacillus mojavensis* strain ifo 15718 with a bootstrap value of 75%, showing high affinity (97.72% of homology) (Figure 2); thus, we determined that strain LY-06 is *Bacillus mojavensis*, named for *Bacillus mojavensis* LY-06 (Figure 3).

### 3.2. Stress Tolerance Detection and Growth Curve of Bacillus mojavensis LY-06 Strain

**Figure 4:**
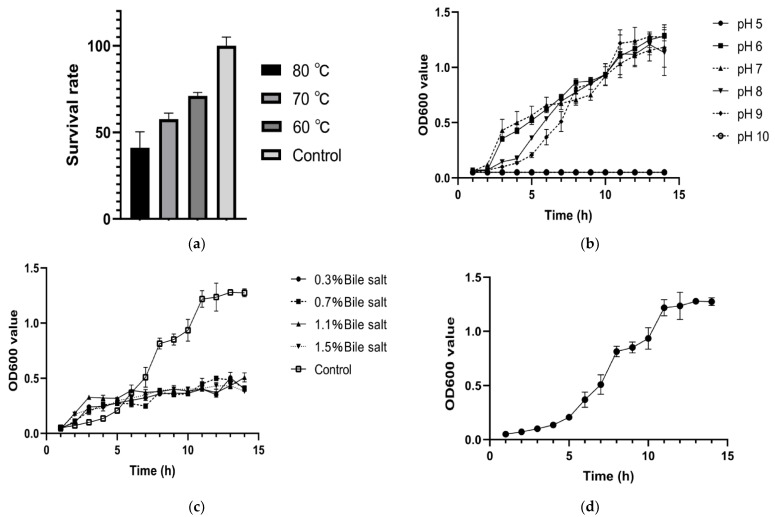
Stress tolerance and growth curve determination of *Bacillus mojavensis* LY-06. (**a**) Temperature tolerance assay of *Bacillus mojavensis* LY-06. The culture medium of the inoculated strains was placed in water baths of different temperatures for 10 min and then cultured at 37 °C for 24 h. The bacterial concentration of each medium was determined, and the bacterial concentration of the medium without high temperature incubation was defined as 100% to calculate the survival rate of each sample. (**b**) pH tolerance assay of *Bacillus mojavensis* LY-06. The strains were inoculated into the mediums of different pH levels, cultivated at 37 °C for 14 h, and the concentration of each medium strain was measured at corresponding time intervals. (**c**) Bile salt tolerance assay of *Bacillus mojavensis* LY-06. The bacterial strains were inoculated into the medium containing different concentrations of bile salts, cultivated at 37 °C for 14 h, and the concentration of each medium strain was measured at corresponding time intervals. (**d**) Growth curve determination of *Bacillus mojavensis* LY-06.

In the production process of live bacteria preparations, granulation and other procedures require high temperature and pressure, which are the key factors restricting the quality of live bacteria preparations. In addition, after oral administration, live bacteria must be digested by digestive juices in the digestive tract before reaching the intestine, thereby exerting a probiotic effect [41]. Therefore, this study determined the acid, bile salt, and temperature tolerance of *Bacillus mojavensis* LY-06 to evaluate the tolerance of *Bacillus mojavensis* LY-06 to different extreme environments during processing and oral administration. Judging from the heat-resistant survival rate, the survival rate of *Bacillus mojavensis* LY-06 still reached 71.7%, 58.2%, and 41.7% after 10 min treatment at 60 °C, 70 ℃, and 80 ℃, respectively, indicating that *Bacillus mojavensis* LY-06 may have good high temperature resistance and may be able to adapt to the high temperature environment generated during the granulation process of live bacteria (Figure 4a). At the same time, *Bacillus mojavensis* LY-06 was sensitive to both acid and alkaline environments: The strain could not grow in LB medium with a pH of less than 6 and a pH of greater than 9 (Figure 4b). We speculate that this may be related to the natural selection of fermenting strains in the neutral pH environment during the fermentation of douchi. Although *Bacillus mojavensis* LY-06 grew relatively slowly in LB medium containing 0.3% bile salts, further increasing the bile salt concentration did not affect the growth of this strain (Figure 4c). Therefore, *Bacillus mojavensis* LY-06 is able to withstand the stimulation of bile salts after entering the digestive tract as an oral probiotic and can exert its probiotic function in the intestinal tract. As a probiotic, although *Bacillus* cannot colonize the digestive tract for a long period of time, it can remain in the digestive tract for a limited period of time; thus, a faster reproduction ability is the basis for ensuring the probiotic activity of *Bacillus* [42]. *Bacillus mojavensis* LY-06 entered the logarithmic growth phase after 4 h of culture. Usually, the fermentation time of *Lactobacillus* and *Bifidobacterium* into the logarithmic growth phase is about 20 h, and the *Bacillus* reported in other studies is also about 12 h, which indicates that the *Bacillus mojavensis* LY-06 screened in this study has a faster growth rate (Figure 4d).

### 3.3. Regulation of Nattokinase Production by Carbon and Nitrogen Sources

**Figure 5:**
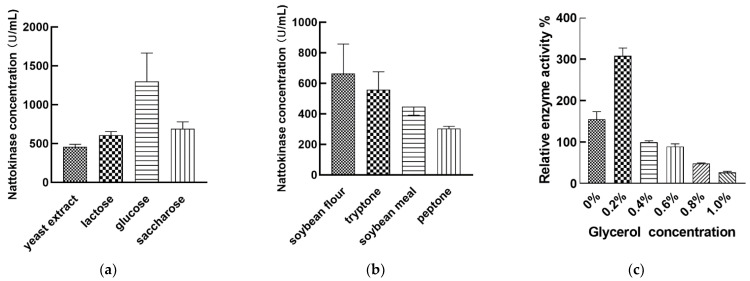
Effects of different types of carbon sources (**a**), nitrogen sources (**b**), and different concentrations of glycerol (**c**) on the production of nattokinase by the fermentation of *Bacillus mojavensis* LY-06.

We evaluated the ability of different carbon sources (yeast extract, lactose, glucose, saccharose) to ferment the *Bacillus mojavensis* LY-06 strain to produce nattokinase and found that glucose was the best carbon source (Figure 5a). Various types of carbon sources such as shrimp shell wastes, maltose, lactose, and galactose have been reported to be the best carbon sources for the fermentation of nattokinase by different *Bacillus* species, and some low-cost carbon sources (tapioca starch) and even industrial wastes (tofu processing wastewater) can also be used as carbon sources for the fermentation of nattokinase [16,26]. We speculate that glucose as a monosaccharide is more easily absorbed and utilized by the *Bacillus mojavensis* LY-06 strain. In this study, soybean flour was considered as the best nitrogen source for nattokinase production by the fermentation of *Bacillus mojavensis* LY-06 strain (Figure 5b). Related studies have reported the effect of different types of nitrogen sources on the yield of nattokinase produced by the fermentation of *Bacillus*. Compared with peptone, beef extract, and tryptone, soybean flour has lower cost and better enzyme production efficiency, so it can be used as a nitrogen source for industrial production [43,44,45]. Adding an appropriate amount of glycerol (6%) to the fermentation broth can effectively increase the cell density of *bacillus subtilis* natto in the fermentation broth, which in turn helps to improve the fermentation yield of nattokinase [46]. In this study, we compared the nattokinase fermentation efficiency of *Bacillus mojavensis* LY-06 with different concentrations of glycerol. The experimental results showed that the best nattokinase fermentation yield was obtained by adding an additional 0.2% glycerol, and the enzyme production rate decreased rapidly above or below 0.2% (Figure 5c). We speculate that the optimal glycerol concentration required for nattokinase fermentation depends not only on the individual differences of *Bacillus*, but also on the ratio of different fermentation media.

### 3.4. Regulation of Nattokinase Production with Inorganic Salt

**Figure 6:**
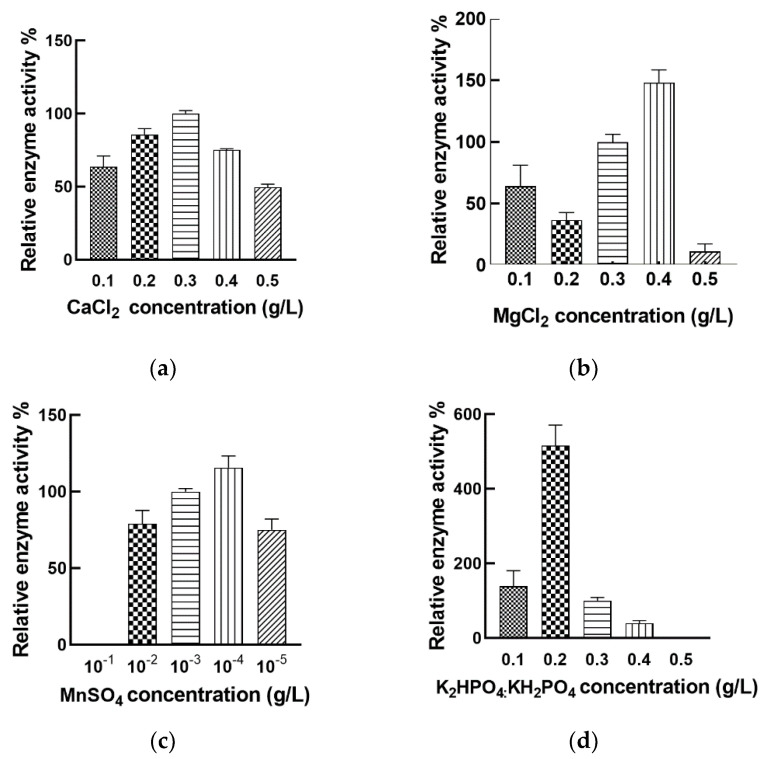
Effects of different concentrations of Ca^2+^ (**a**), Mg^2+^ (**b**), Mn^2+^ (**c**), and K_2_HPO_4_:KH_2_PO_4_ (**d**) on the production of nattokinase by the fermentation of *Bacillus mojavensis* LY-06.

Inorganic salts are important trace components and participate in the regulation of microbial metabolic processes. Some microorganisms require Ca^2+^ to synthesize proteases, and Ca^2+^ as a metal activator plays a key role in the thrombolytic activity of nattokinase [47]. We explored the effect of different Ca^2+^ concentrations on the fermentation of *Bacillus mojavensis* LY-06 to produce nattokinase. Our study showed that adding 0.3 g/L of Ca^2+^ could effectively improve the fermentation yield of nattokinase (Figure 6a). Jo found that increasing the Ca^2+^ concentration of the fermentation broth could increase the yield of *Bacillus licheniformis* CH 3-17-derived nattokinase (AprE 3-17) [48]. However, Kotb’s research showed that excessive Ca^2+^ concentration can inhibit the production of nattokinase derived from *Bacillus megaterium* KSK-07. Therefore, the appropriate Ca^2+^ concentration in the fermentation broth is the key to improve the yield of *Bacillus* nattokinase.

Mg^2+^ is involved in the oxidation of microbial substances; it also affects protein synthesis and is an activator of many enzymes [44]. In this study, the best nattokinase fermentation yield was obtained by adding 0.4 g/L MgCl_2_ to the nattokinase fermentation broth, while the yield decreased rapidly when MgCl_2_ was higher than 0.4 g/L (Figure 6b). The currently reported optimal Mg^2+^ concentration of nattokinase produced by *Bacillus* is between 0.02% and 0.7%, and the optimal Mg^2+^ concentration in our fermentation system is relative smaller than some reported concentrations [18,45]. 

We found that the proper addition of Mn^2+^ (10^−3^ g/L) could increase the fermentable amount of nattokinase in *Bacillus mojavensis* LY-06 (Figure 6c). This is in contrast to the experimental results of Man et al. [44]. Since the trace amount of Mn^2+^ in *Bacillus* facilitates its absorption of phosphorus elements and the formation of spores, we speculate that the change of Mn^2+^ concentration in the fermentation broth has an important influence on the synthesis of nattokinase in *Bacillus*.

Interestingly, the concentration of K^+^ in the fermentation broth had a great influence on the nattokinase yield of *Bacillus mojavensis* LY-06. (Figure 6d). When the K^+^ concentration in the fermentation broth was 0.2 g/L, the maximum nattokinase yield can be obtained; however, using fermentation broths containing 0.1 g/L and 0.3 g/L K^+^ for fermentation, the enzyme yields were only 26.9% and 19.3% of the optimal yields, respectively. We speculate that high concentration of K^+^ changes the cell permeability of *Bacillus*, which indirectly affects the production of nattokinase, while too low a concentration of K^+^ affects its normal metabolic process.

### 3.5. Effect of Orthogonal Array Design of Nutrient Optimization on Nattokinase Production

Based on the above results, glycerol, K_2_HPO_4_:KH_2_PO_4_, MnSO_4_, and CaCl_2_ were chosen for the orthogonal array experiment, and Minitab 16 was applied for the statistical analysis of the fermentation data. According to our results, MnSO_4_ was found to be the most significant factor, followed by glycerol, K_2_HPO_4_:KH_2_PO_4_, and CaCl_2_ (Table 2). The optimal concentrations of MnSO_4_, glycerol, K_2_HPO_4_:KH_2_PO_4_, and CaCl_2_ for high level nattokinase production were 10^−5^ mol/L, 0.4%, 0.1 g/L and 0.3 g/L, respectively. The optimum enzyme yield under this fermentation condition was 1434.64 U/mL.

### 3.6. Cloning, Expression, and Purification of rAprY

**Figure 7:**
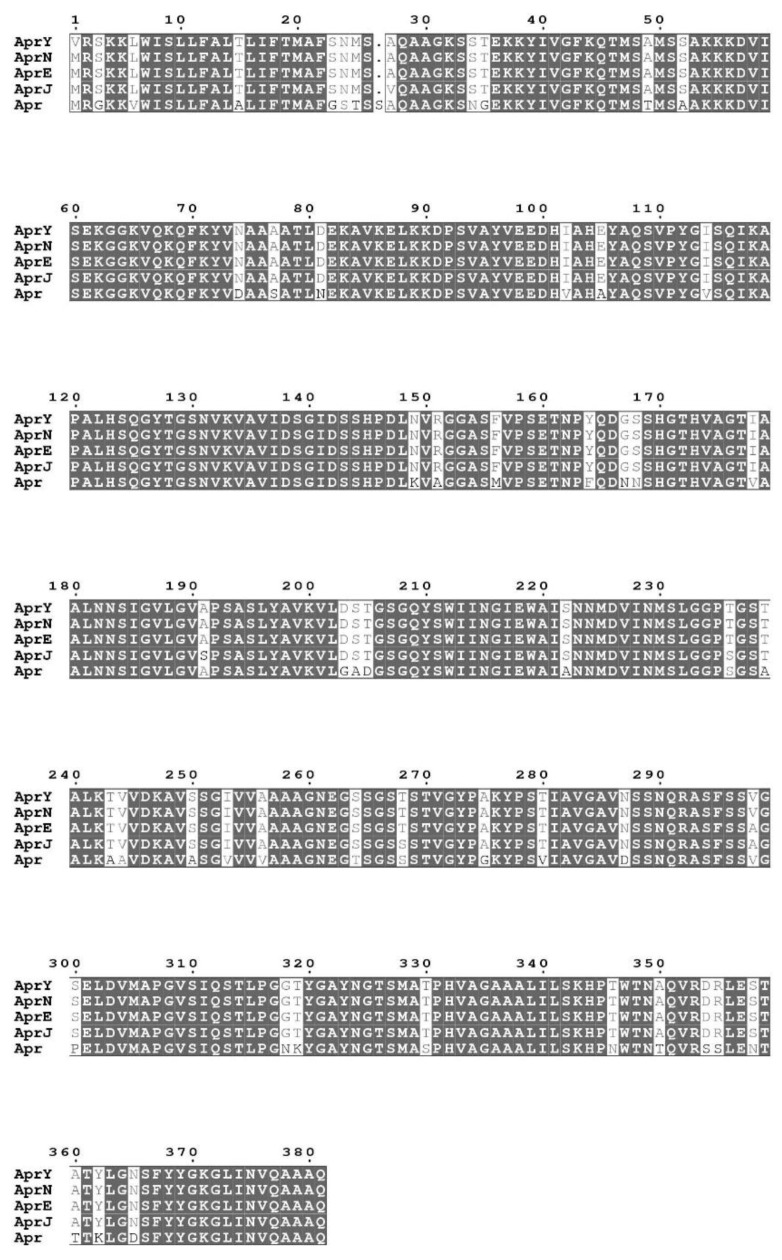
Multiple sequence alignment of *Bacillus mojavensis* LY-06 nattokinase (LY-06) compared with other serine proteases homologs. AprN: subtilisin NAT; AprE: subtilisin E; AprJ: subtilisin BPN’; Apr: subtilisin BPN’.

We used the nattokinase encoding gene derived from *Bacillus* natto (*aprN*, EC:3.4.21.62) as a template to design primers, and successfully amplified the complete nattokinase encoding gene sequence of *Bacillus mojavensis* LY-06 (*aprY*). The *aprY* gene had a 1146 bp open reading frame that encoded a protein of 381 residues, including a signal peptide of 29 amino acids, a propeptide of 77 amino acids, and a mature peptide of 275 amino acids with the molecular weight of 27.7 kDa. In silico analysis showed that AprY had 99.7%, 99.5%, 98.4%, and 86.4% sequence homology with subtilisin NAT, subtilisin E, subtilisin J, and subtilisin BPN’, which belong to the same alkaline serine protease family (Figure 7). Although only a few amino acid residues are varied from the reported sequences of alkaline serine proteases, their kinetic parameters, substrate specificity, and other properties are quite different. Nattokinase is the only alkaline serine protease widely reported to have thrombolytic activity, which may be due to the spatial conformational difference between nattokinase and other proteases in the same family [10]. The AprY sequence obtained in this study has the highest homology with the reported nattokinase AprN sequence, indicating that AprY also has thrombolytic activity. 

**Figure 8:**
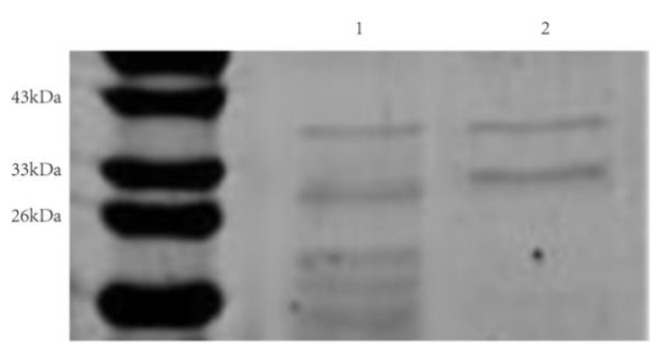
SDS-PAGE of purified AprY and rAprY expressed in *Escherichia coli* BL21. Lanes 1–2 represent partially purified AprY, rAprY purified by the Ni-NTA column and the DEAE Sepharose Fast Flow column, respectively.

Using engineered bacteria to express nattokinase has been proven to be an important way to increase the yield of nattokinase. At present, nattokinase has been expressed in *Escherichia coli* [27], *Lactobacillus* [28], *Bacillus* [29], and *Pichia pastoris* [30]. However, the induced expression of nattokinase in *Escherichia coli* often forms inactive inclusion bodies; therefore, how to achieve the soluble expression of the nattokinase-encoding gene in *Escherichia coli* has become an urgent problem to be solved [27]. In this study, the complete nattokinase gene *aprY* (signal peptide + propeptide + mature peptide) of the 9 *Bacillus mojavensis* LY-06 strain was constructed into plasmid peT28a(+), and the supernatant expression and purification of recombinant AprY (rAprY) in *Escherichia coli* were realized by adding an additional His-tag to the C-terminus of the AprY protein. The results of rAprY purification showed two bands, in which the band at 29 kDa was consistent with the size of the mature peptide of rAprY, and the other band, with a size of about 36 kDa, was presumed to be rAprY containing the propeptide (Figure 8). The purified rAprY exhibited fibrinolytic activity, confirming the inference that the propeptide of nattokinase may be involved in the correct folding of nattokinase as an intramolecular chaperone [49]. Although reducing the induction temperature and lowering the concentration of the inducer can increase the supernatant expression of nattokinase in *Escherichia coli*, the supernatant expression of rAprY was not high in this study. Therefore, the optimization of the conditions for recombinant nattokinase expression in *Escherichia coli* remains to be further explored.

**Figure 9:**
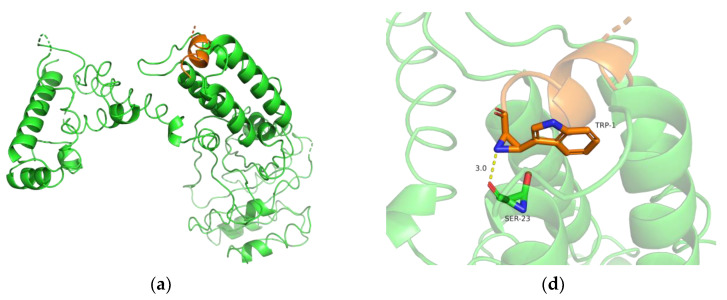

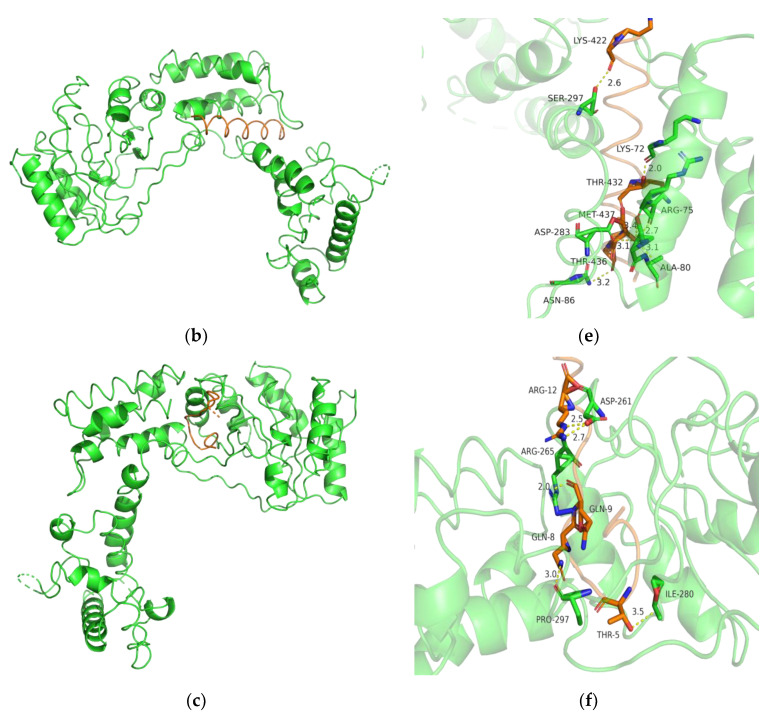
Molecular docking of different signal peptides with *Escherichia coli* SRP. SRP and signal peptides are shown as cartoons colored in green and orange, respectively, and the related residues are shown as stick models. The spatial structures of the *Escherichia coli* DsbA signal peptide (**a**), the nattokinase signal peptide (**b**), and the nattokinase signal peptide containing the upstream sequence of peT28a(+) (**c**) docking with SRP are shown in the left picture; the interactions between the *Escherichia coli* DsbA signal peptide (**d**), the nattokinase signal peptide (**e**), the nattokinase signal peptide containing the upstream sequence of peT28a(+) (**f**) and SRP are shown in the picture on the right.

In the *Escherichia coli* expression system, the nattokinase precursor protein relies on a co-translational transport pathway for secretion [47]. In this pathway, signal particle recognition (SRP) targets the precursor nattokinase to the SecYEG translocation pore by binding to the signal peptide of nattokinase and the SRP protein receptor FtsY, secreting the synthesized precursor protein out of the bacteria for correct protein folding [50]. The open reading frame of the peT28a(+)-aprY vector constructed in this study contains the signal peptide sequence of *Bacillus* and its upstream His tag and T7 tag; thus, we attempted to molecularly dock this upstream tagged signal peptide and nattokinase natural signal peptide with SRP, respectively, to explain the low soluble expression of rAprY in *Escherichia coli*. As a control, both the DsbA signal peptide derived from *Escherichia coli* (a signal peptide belonging to the co-translational transport pathway of the *Escherichia coli* SecB secretion system) (Figure 9a,d) and the *Bacillus* nattokinase signal peptide (Figure 9b,e) could correctly dock with SRP. Compared with the natural signal peptide of nattokinase, the His tag and T7 tag of the upstream sequence of peT28a(+) form 5 hydrogen bonds with SRP, so that the downstream *Bacillus* signal peptide sequence cannot interact with SRP, which eventually leads to docking failure (Figure 9c,f). Therefore, we speculate that in the open reading frame of peT28a(+)-aprY, the expressed His tag and T7 tag upstream of the natural nattokinase signal peptide may interfere with the interaction between the signal peptide and SRP, resulting in the failure of nattokinase precursor protein to be secreted and folded correctly, thereby forming an insoluble inclusion body.

### 3.7. Biochemical Characterization and Productivity Curves of AprY and rAprY (C-Terminal Contains His-Tag)

**Figure 10:**
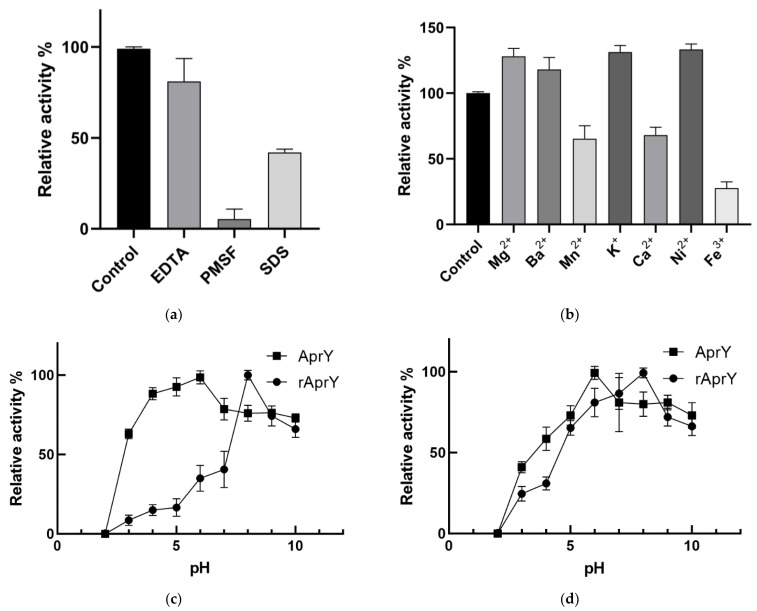

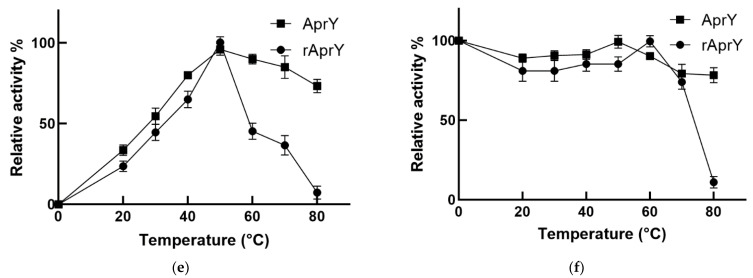
Characterization and enzymatic properties of rAprY and AprY. Average values from three independent experiments are presented, and all of the measurements of the enzyme activities were measured in three parallels. (**a**) Effects of different inhibitors on fibrinolytic activity of rAprY; (**b**) effects of different salt ions on the fibrinolytic activity of rAprY; (**c**) optimum pH of rAprY and AprY; (**d**) pH stability of rAprY and AprY. The residual fibrinolytic activity of the enzymes was determined after 30 min incubation of the enzymes in different pH solutions. The highest enzyme activity was defined as 100%. (**e**) Optimum temperature of rAprY and AprY; (**f**) temperature stability of rAprY and AprY. The residual fibrinolytic activity of the enzymes was determined after incubating the enzymes in water baths of different temperatures for 30 min. The highest enzyme activity was defined as 100%.

In this study, the inhibitor specificity of rAprY with His-tag at C-terminus was first determined (Figure 10a). rAprY was completely inhibited by PMSF, which is a specific inhibitor of some alkaline serine protease families, including nattokinase [51]. The fibrinolytic activity of rAprY was slightly inhibited by EDTA, which was similar to nattokinase derived from *bacillus subtilis* VITMS 2 [52]. Since Ca^2+^ is a metal activator of nattokinase, it is speculated that EDTA reduces the Ca^2+^ concentration in the enzyme reaction system through the complexation reaction to affect the fibrinolytic activity of rAprY. Interestingly, adding the anionic surfactant SDS to the reaction system also reduced the fibrinolytic activity of rAprY. This phenomenon has also been reported in nattokinase derived from *Bacillus subtilis* strain VTCC-DVN-12-01 [29]. Different inorganic salts have different effects on the enzymatic activity of rAprY (Figure 10b): adding 5 mM Mg^2+^, Ba^2+^, K^+^, and Ni^2+^ to the enzyme reaction system enhanced the thrombolytic activity of rAprY, while Mn^2+^, Ca^2+^, and Fe^3+^ inhibited the enzyme, to varying degrees. 

Since Nakamura first obtained the nattokinase gene *aprN,* many research papers have described the enzymatic properties of nattokinase from different microbial sources [53]. Nattokinase is a neutral enzyme with relatively stable enzymatic activity in the range of 40–65 °C, and is rapidly inactivated under acidic conditions [7,19,44]. This study compared the enzymatic properties of AprY and rAprY, and found that the optimum pH (pH = 8) of rAprY was higher than that of AprY (pH = 6) (Figure 10c). Furthermore, the enzymatic activity of rAprY treated at pH 4 for 30 min is only about 20% of that at pH 8, while the corresponding AprY has higher pH stability (greater than 50%) (Figure 10d). Different from the optimum pH and its stability, the optimum temperature of rAprY was the same as that of AprY (50 °C), but the enzyme activity decreased faster with the increase in temperature (Figure 10e); after incubation at 80 °C for 30 min, AprY still retained about 75% of the enzymatic activity, while rAprY was inactivated, indicating that rAprY has relatively lower thermal stability (Figure 10f).

**Figure 11:**
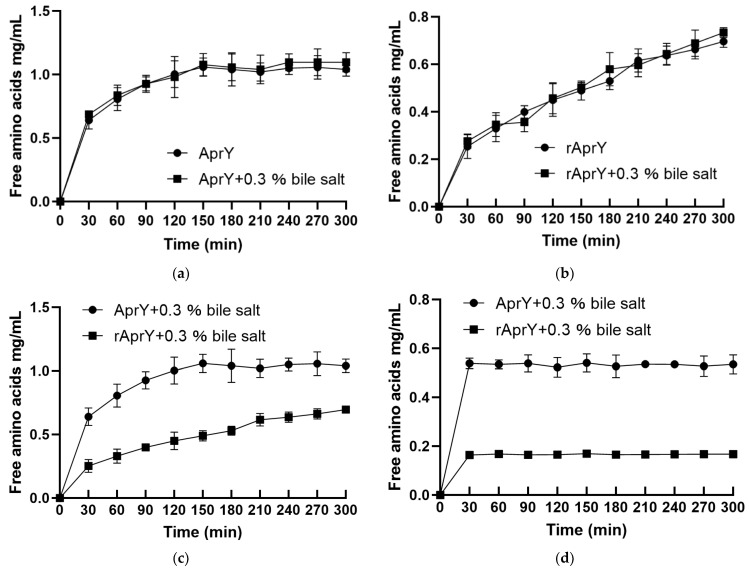
Productivity curves for AprY and rAprY. Upper two figures: the effect of 0.3% bile salt on the productivity curves of AprY (**a**) and rAprY (**b**) under their respective optimal catalytic conditions (for AprY, 50 °C, pH 6; for rAprY, 50 °C, pH 8). Lower two figures: comparison of productivity curves of AprY and rAprY at 50 °C (**c**) and 75 °C (**d**).

Enzymatic productivity is a measurement of product formation or substrate disappearance over time at a prescribed temperature under specified reaction conditions; it provides an accurate measurement of the durability and reaction yield of enzymatic processes [54]. This study investigated the productivity curves at optimum catalysis conditions (50 °C) and high temperature (75 °C), in the absence and presence of bile salts, for both AprY and rAprY. After incubation for 300 min in the reaction system containing 0.3% bile salts, under optimal reaction conditions, 0.3% bile salts did not affect the ability of AprY and rAprY to hydrolyze fibrin to generate free amino acids, indicating that both of them can maintain the fibrinolytic activity in the bile salt-containing digestive tract system. This is the first time we have determined the effect of bile salts on the fibrinolytic activity of nattokinase (Figure 11a,b). To explore the effect of high temperature on the productivity of AprY and rAprY, we compared the productivity curve of the two enzymes at 50 °C and 75 °C. The results showed that both AprY and rAprY were rapidly inactivated at 75 °C; the free amino acids production of AprY and rAprY after 300 min incubation at 75 °C was only 51.4% and 22.9% of that at 50 °C, respectively (Figure 11c,d). Notably, under any condition, AprY had much higher productivity than rAprY. The lower productivity of rAprY may be related to its lower stability, causing it to unfold earlier than AprY during enzymatic reactions.

It has been reported that recombinant nattokinase expressed in *Escherichia coli* has lower fibrinolytic activity than natural nattokinase, and there is a lack of comparative studies on nattokinase expressed by these two hosts [47]. In order to facilitate the purification and identification process of recombinant proteins expressed in *Escherichia coli*, many studies have adopted methods such as adding protein purification tag sequences and protein detection tag sequences at the end of the protein coding sequence [49]. We speculate that the insertion of these tags may have an impact on the enzymatic properties of the recombinant proteins. 

**Figure 12:**
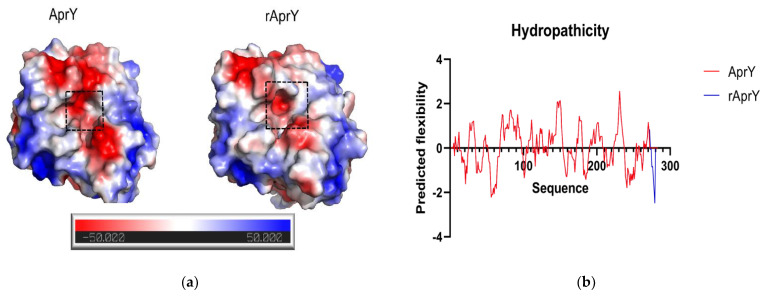

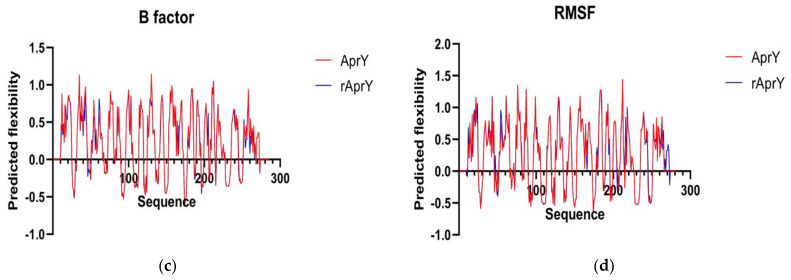
Analysis of surface charge (**a**), hydrophobicity (**b**), b-factor value (**c**), and RMSF value (**d**) of AprY and rAprY. In the hydrophobicity analysis, the higher the predictive value of the amino acid residue, the stronger the hydrophobicity.

To elucidate the differences between rAprY and AprY in the enzymatic properties and productivity, the protein structure of rAprY was first predicted using I-Tasser online software, and the differences in intra-protein interactions between rAprY and AprY were predicted using a variety of bioinformatics methods. After calculation of internal interactions, we found that the isoelectric point of rAprY and the pKa value of key amino acid residues in its active center are lower than those of AprY (Table 3). Different from the predicted decrease in isoelectric point value of rAprY, the non-denaturing PAGE results showed that the electrophoresis rate of rAprY containing His-tag at the C-terminus was relatively slower than that of AprY, and its apparent molecular weight was slightly larger than the actual molecular weight. Compared with AprY, the surface charge of rAprY also changed accordingly: the negative charge distribution in the active center and its periphery of rAprY were reduced (Figure 12a). Previous studies have shown that the negative charge would neutralize the positive repulsion of the protonated residue at low pH, thus favoring the enzyme acidic stability [55]. Therefore, mutating basic amino acid residues on the surface and active center of proteins to negatively charged acidic amino acid residues has been shown to improve the acidic stability of proteins [56]. The increase in the hydrophilicity of the protein surface is also related to its acid stability [55]. However, no significant difference was found in the surface hydrophobicity analysis of the two nattokinases (Figure 12b). In summary, we believe that the positively charged His-tag at the C-terminus of rAprY improves its optimal pH and reduces its acidic stability by changing the protein isoelectric point and reducing the negative charge on the enzyme surface. 

We found that the fibrinolytic activity of rAprY was lower than that of AprY. By comparing the number of hydrogen bonds of the two enzymes, we found that rAprY (284) has more hydrogen bonds than AprY (260). In general, the increase in hydrogen bonds within the enzyme will increase the rigidity of the enzyme, thereby reducing the catalytic activity of the enzyme [57]. At higher temperatures, the thermal stability of rAprY decreases rapidly compared to AprY. Although the number of hydrogen bonds of rAprY is increased, its stability also depends on whether the entire structure is flexible (global flexibility) or only the regions surrounding the active site (local flexibility). In this study, the B-factor values of amino acid residues in multiple loop regions of rAprY were higher than those of the corresponding AprY, suggesting that the more flexible loop region of rAprY is the reason for its instability at high temperatures (Figure 12c) [58]. The RMSF analysis of AprY and rAprY also supports the above judgment. We found that compared with AprY, the 19–20 (loop), 46–47 (β-sheet) and 216–218 (loop) positions of rAprY have a higher RMSF, indicating that the highly flexible loop of rAprY affects its stability at high temperatures (Figure 12d) [59].

The modification of enzymes through protein engineering is an effective means to improve the performance of enzymes. At present, many literatures have carried out molecular modification on the defects of nattokinase, such as improving fibrinolytic activity [60], improving acid stability [61] and antioxidant capacity [49]. The research data on the heterologous expression and enzymatic properties of *Bacillus mojavensis* LY-06 nattokinase provide important theoretical support for the future molecular modification of the enzyme.

## 4. Conclusions

Improving the oral efficacy of nattokinase and probiotics expressed nattokinase is crucial for promoting the market application of nattokinase. In this study, a bile salt-tolerant strain of *Bacillus mojavensis* LY-06 was isolated from douchi, and the stress tolerance and fermentation process of the strain were studied. We achieved the soluble expression of nattokinase derived from *Bacillus mojavensis* LY-06 in *Escherichia coli* and compared the enzymatic properties of recombinant nattokinase and natural nattokinase. The difference in productivity between the two enzymes under bile salt-containing conditions and high temperature conditions was also analyzed. Finally, we explained the poor activity and stability of *Escherichia coli*-expressed recombinant nattokinase by bioinformatics analysis.

## Figures and Tables

**Table 1:** Orthogonal test design of the AprY fermentation process.

	A Glycerol (%)	B K_2_HPO_4_:KH_2_PO_4_	C MnSO_4_ (mol/L)	D CaCl_2_ (g/L)
1	0	0.1	10^−3^	0.2
2	0.2	0.2	10^−4^	0.3
3	0.4	0.3	10^−5^	0.4

**Table 2:** Experimental results and analysis of L9 (34) orthogonal experiments for Nattokinase Production.

No.	Variable				Nattokinase Yield (U/mL)
	A: Glycerol (%)	B: K_2_HPO_4_:KH_2_PO_4_ (g/L)	C: MnSO_4_ (mol/L)	D: CaCl_2_ (g/L)	
1	A1	B1	C1	D1	541.28
2	A1	B2	C2	D2	240.84
3	A1	B3	C3	D3	931.81
4	A2	B1	C2	D3	484.80
5	A2	B2	C3	D1	619.29
6	A2	B3	C1	D2	234.17
7	A3	B1	C3	D2	1434.64
8	A3	B2	C1	D3	1030.80
9	A3	B3	C2	D1	512.51
K1	571.31	820.24	602.08	557.69	
K2	446.09	630.31	412.72	636.55	
K3	992.65	481.44	995.25	815.80	
R	546.56	338.80	582.53	258.11	
Optimal level	A3	B1	C3	D2	

**Table 3:** Internal interactions of AprY and rAprY (C-terminal contains His-tag).

Enzyme	AprY	rAprY
isoelectric point	6.97	6.61
pKa of Asp 32	3.5	<0
pKa of His 64	7.7	7.8
salt bridges	52	51
H bonds	260	284

## Data Availability

The data presented in this study are available on request from the corresponding author.
